# Lower-Extremity Injuries Predominate in American High School Tennis Players

**DOI:** 10.1016/j.asmr.2023.100811

**Published:** 2023-10-19

**Authors:** Aaron C. Llanes, David G. Deckey, Nan Zhang, Kara L. Curley, Natalie D. Curley, Anikar Chhabra, Matthew T. Neal

**Affiliations:** a University of Arizona College of Medicine, Phoenix, Arizona, U.S.A.; b Department of Orthopaedic Surgery, Mayo Clinic Hospital, Phoenix, Arizona, U.S.A.; c Department of Quantitative Health Sciences, Mayo Clinic, Scottsdale, Arizona, U.S.A.; d Department of Neurologic Surgery, Mayo Clinic Hospital, Phoenix, Arizona, U.S.A.; e Midwestern University School of Medicine, Glendale, Arizona, U.S.A.; f Department of Neurosurgery and Spine, Roper St. Francis, Charleston, South Carolina, U.S.A.

## Abstract

**Purpose:**

To describe injury epidemiology in U.S. adolescent tennis players between 2014 and 2018 via the High School Reporting Information Online (HS RIO) database.

**Methods:**

The HS RIO database was queried for injury data on high school tennis players as reported by athletic trainers between 2014 and 2018. Injuries were analyzed according to athlete demographics, injury type, location, and context. Variables of interest between male and female athletes were compared using Pearson χ^2^ test or Fisher exact test.

**Results:**

In total, 176 injuries in high school tennis players between 2014 and 2018 were identified in the HS RIO database. Overall, 25.6% (45/176) occurred in the ankle, 12.5% (22/176) in the knee, and 9.7% (17/176) in the wrist. The most common types of injuries were ligament sprains and muscle strains at 35.2% (62/176) and 17.6% (31/176) of injuries, respectively. Although most injuries were unrelated to contact, such as overuse and heat exertion or stroke, 28.7% (47/176) of injuries were the result of rotation around a planted foot/inversion of the foot. We found no difference in injury patterns between male and female high school tennis athletes.

**Conclusions:**

We found no difference in injury patterns between male and female U.S. high school tennis athletes. The ankles, knees, and wrists were the most commonly injured areas in this population. The most common types of injuries were ligament sprains and muscle strains. Although many injuries were new, athletes rarely required surgery and returned to play. Finally, we found no difference in injury patterns between male and female high school tennis athletes.

**Clinical Relevance:**

The epidemiology of injuries among high school tennis players is poorly understood. The information from this study will help us to understand these injuries and how we may be able to better prevent them.

The International Tennis Federation estimates that more than 87 million people across the world play tennis as of 2021, an increase of 4.5% compared with 2018.^1^ Participation levels vary globally, from recreational to professional and across different categories including men’s, women’s, doubles, and wheelchair tennis. A snapshot of tennis participation shows 7,275 ranked players in International Tennis Federation Junior World Tennis Tour and 3,619 professional players with an Association of Tennis Professionals or Women’s Tennis Association ranking.^1^

Tennis requires a balance of agility, flexibility, and power that is distinct from other racquet sports.^2^ Proper technique delivered through a kinetic chain traveling from the ground through the lower limbs, trunk, upper extremity, and into the racquet drives the tennis ball across the court.^3^^,^^4^ In addition, tennis players must navigate the court effectively, running, stopping, and changing direction at a moment’s notice. Therefore, the forces needed to perform these strokes and movements place considerable strain on the tennis players’ musculoskeletal system, oftentimes leading to injury.^5^^,^^6^

Previous studies have described the epidemiology of injuries in tennis players. In a meta-analysis, Pluim et al.^7^ concluded that injuries in tennis players were most common in the lower extremities, followed by the upper extremities, and then finally the trunk. In a review, Dines et al.^8^ stated that most lower-extremity injuries were acute and included sprains or strains, whereas most upper-extremity injuries were chronic, particularly elbow tendinopathies. Although injury rates across levels of competition, sex, and age have been compared, the reports have been inconsistent.^2^^,^^7^^,^^9^

To date, most studies in the literature on tennis injury epidemiology have focused on a single cohort of competitors or particular demographic.^10^^,^^11^ For instance, a previous survey of injuries using the National Collegiate Athletic Association Injury Surveillance Program only focused on U.S. men’s and women’s college tennis and found similar injury rates overall between men and women.^12^ Another survey by Hutchinson et al.^9^ focused solely on injuries within United States Tennis Association’s boys’ tennis. However, a review of the literature reveals a gap in injury epidemiology for high school tennis players within the United States.

Given that these individuals are still physically growing and mentally maturing, high school athletes can be particularly vulnerable to injury.^13^ Previous studies on adolescent injury epidemiology attribute a majority of adolescent injuries to overuse, risky behavior, and instability from rapid musculoskeletal development.^14^, ^15^, ^16^ Although these studies have focused more on major sports such as basketball, soccer, football, baseball, and softball, injury epidemiology for U.S. adolescent tennis athletes also should be analyzed to help inform coaches, trainers, parents, and players on injury-prevention strategies.^17^ This study uses data from the High School Reporting Information Online (HS RIO) database to report injuries suffered by U.S. high school tennis athletes.

The purpose of this study is to describe injury epidemiology in U.S. adolescent tennis players between 2014 and 2018 via the HS RIO database. We hypothesized that high school tennis athletes would experience mostly lower-extremity injuries, similar to other populations of tennis players.

## Methods

### Data Collection

The HS RIO database was queried to collect data on injuries in U.S. high school tennis players from 2014 to 2018 from more than 100 participating high school across the United States. The HS RIO has been validated and methods of data collection have been described.^18^

The HS RIO database monitors injuries and athletic exposures described as school-sanctioned practices and competitions as reported by each institution’s athletic trainer(s) (AT). Each report contains information on the injured athlete, including athlete demographics, injury, and injury context. Athlete demographics contain information about the athlete’s age, height, weight, sex, and school year. Injury information refers to the diagnosis, severity, and anatomical location. Injury context refers to the situation in which the injury occurred, such as during practice or competition, preseason, or postseason and other sport idiosyncrasies. This study was reviewed and granted an institutional review board exemption.

### Data Analysis

The categorical variables are summarized as frequency (percentage). The variables of interest were compared between male athletes and female athletes using Pearson χ^2^ test or Fisher exact test when appropriate. All statistical analyses were conducted using RStudio software (RStudio 4.0.3; PBC, Boston, MA) and 0.05 was chosen as the cut-off criterion for statistical significance.

## Results

### Injury by Year and Level of Play

A total of 176 injuries in high school tennis players were identified in the HS RIO database between 2014 and 2018. A breakdown of injuries by year shows 35 reported injuries in the 2014/2015 season, 47 in the 2015/1016 season, 41 in the 2016/2017 season, and 53 in the 2017/2018 season (Appendix Table 1, available at www.arthroscopyjournal.org). Of the injuries reported, 82.4% (145/176) of the injured athletes played at the varsity level, 13.1% (23/176) played at the junior varsity level, and 0.6% (1/176) at the freshman level (Appendix Table 1).

### Principal Body Part Injured, Injury Type, and Mechanism of Injury

Overall, 25.6% (45/176) occurred in the ankle, 12.5% (22/176) in the knee, and 9.7% (17/176) in the wrist. Injuries to the eyes, nose, and upper arm were recorded, but rare at 0.6% (1/176) of injuries (Table 1). The most common types of injuries were ligament sprains and muscle strains at 35.2% (62/176) and 17.6% (31/176) of injuries, respectively. Acute cardiac events, abrasions, stress fractures and avulsions were noted, but rare at 0.6% (1/176) of injuries (Table 2). Regarding the general mechanism of injury, 44% (77/176) of injuries occurred acutely and without contact, whereas 24.0% (42/176) were considered chronic. Analysis of tennis-specific mechanisms of injury reiterated that although most injuries were unrelated to contact such as overuse and heat exertion or stroke, 28.7% (47/176) of injuries were the result of rotation around a planted foot/inversion of the foot (Table 3).

**Table 1:** Injury by Body Part

	Male (N = 68)	Female (N = 108)	Total (N = 176)	*P* Value
Head				.691∗
Head/face	3 (4.4%)	4 (3.7%)	7 (4.0%)	
Eyes	0 (0.0%)	1 (0.9%)	1 (0.6%)	
Nose	0 (0.0%)	1 (0.9%)	1 (0.6%)	
Trunk				
Chest/thoracic-spine/ribs	1 (1.5%)	1 (0.9%)	2 (1.1%)	
Lower back/lumbar spine/pelvis	5 (7.4%)	5 (4.6%)	10 (5.7%)	
Upper extremity				
Shoulder	4 (5.9%)	10 (9.3%)	14 (8.0%)	
Upper arm	1 (1.5%)	0 (0.0%)	1 (0.6%)	
Elbow	3 (4.4%)	3 (2.8%)	6 (3.4%)	
Forearm	3 (4.4%)	0 (0.0%)	3 (1.7%)	
Wrist	5 (7.4%)	12 (11.1%)	17 (9.7%)	
Hand/finger/thumb	1 (1.5%)	1 (0.9%)	2 (1.1%)	
Lower extremity				
Hip	1 (1.5%)	4 (3.7%)	5 (2.8%)	
Thigh/upper leg	3 (4.4%)	5 (4.6%)	8 (4.5%)	
Knee	8 (11.8%)	14 (13.0%)	22 (12.5%)	
Lower leg	5 (7.4%)	9 (8.3%)	14 (8.0%)	
Ankle	17 (25.0%)	28 (25.9%)	45 (25.6%)	
Foot/toe	8 (11.8%)	4 (3.7%)	12 (6.8%)	
Other	0 (0.0%)	3 (2.8%)	3 (1.7%)	

∗ Fisher exact test for count data.

**Table 2:** Injury Type

	Male (N = 68)	Female (N = 108)	Total (N = 176)	*P* Value
Primary type of injury				.905∗
Abrasion	0 (0.0%)	1 (0.9%)	1 (0.6%)	
Stress fracture	0 (0.0%)	1 (0.9%)	1 (0.6%)	
Tendonitis	7 (10.3%)	10 (9.3%)	17 (9.7%)	
Torn cartilage	0 (0.0%)	2 (1.9%)	2 (1.1%)	
Other	3 (4.4%)	8 (7.4%)	11 (6.2%)	
Avulsion	0 (0.0%)	1 (0.9%)	1 (0.6%)	
Subluxation	2 (2.9%)	3 (2.8%)	5 (2.8%)	
Ligament sprain	24 (35.3%)	38 (35.2%)	62 (35.2%)	
Bursitis	1 (1.5%)	1 (0.9%)	2 (1.1%)	
Muscle strain	11 (16.2%)	20 (18.5%)	31 (17.6%)	
Tendon strain	1 (1.5%)	3 (2.8%)	4 (2.3%)	
Cardiac event (acute)	0 (0.0%)	1 (0.9%)	1 (0.6%)	
Apophysitis	2 (2.9%)	0 (0.0%)	2 (1.1%)	
Shin splints	3 (4.4%)	1 (0.9%)	4 (2.3%)	
Heat illness/injury	0 (0.0%)	2 (1.9%)	2 (1.1%)	
Concussion	3 (4.4%)	4 (3.7%)	7 (4.0%)	
Contusion	5 (7.4%)	4 (3.7%)	9 (5.1%)	
Dislocation	1 (1.5%)	2 (1.9%)	3 (1.7%)	
Fracture	5 (7.4%)	6 (5.6%)	11 (6.2%)	

∗ Fisher exact test for count data.

**Table 3:** Injury Mechanisms

	Male (N = 68)	Female (N = 108)	Total (N = 176)	*P* Value
Basic injury mechanism				.085∗
N-Miss	0	1	1	
Contact with another person	1 (1.5%)	0 (0.0%)	1 (0.6%)	
Contact with playing surface	16 (23.5%)	15 (14.0%)	31 (17.7%)	
Contact with playing apparatus	3 (4.4%)	13 (12.1%)	16 (9.1%)	
Acute no contact	29 (42.6%)	48 (44.9%)	77 (44.0%)	
Overuse/chronic	16 (23.5%)	26 (24.3%)	42 (24.0%)	
Illness (eg, heat illness, skin infection, asthma, etc)	0 (0.0%)	4 (3.7%)	4 (2.3%)	
Other	1 (1.5%)	0 (0.0%)	1 (0.6%)	
Unknown	2 (2.9%)	1 (0.9%)	3 (1.7%)	
Tennis-specific mechanism of injury				.092∗
N-Miss	5	7	12	
Struck by ball	0 (0.0%)	3 (3.0%)	3 (1.8%)	
Other	6 (9.5%)	7 (6.9%)	13 (7.9%)	
Unknown	4 (6.3%)	3 (3.0%)	7 (4.3%)	
Stepped or tripped on ball	0 (0.0%)	6 (5.9%)	6 (3.7%)	
Contact with racquet	1 (1.6%)	5 (5.0%)	6 (3.7%)	
Contact with playing surface	9 (14.3%)	10 (9.9%)	19 (11.6%)	
Contact with net	1 (1.6%)	0 (0.0%)	1 (0.6%)	
Contact with another player	1 (1.6%)	0 (0.0%)	1 (0.6%)	
Contact with out of bounds object	1 (1.6%)	1 (1.0%)	2 (1.2%)	
Rotation around a planted foot/inversion	22 (34.9%)	25 (24.8%)	47 (28.7%)	
Other noncontact (overuse, heat, etc)	18 (28.6%)	41 (40.6%)	59 (36.0%)	

∗ Fisher exact test for count data.

### Incidence, Time of Tennis Season, and Context of Injury

Overall, 85.6% (149/176) injuries were new, acute injuries, whereas 14.4% (25/176) of injuries were recurring or chronic (Appendix Table 1). When we analyzed the season of play, 75.4% (132/176) of injuries occurred in the regular season, whereas 22.3% (39/176) of injuries occurred in the preseason (Appendix Table 1). Analysis of injuries by athlete exposure showed that 43.8% (77/176) of injuries occurred in competition, compared with 56.2% (99/176) in practice. For the injuries that occurred during competition, 34.8% (24/176) occurred in the second set and 17.4% (12/176) in the first set. For injuries that occurred during practice, 42.7% (41/176) occurred 1 to 2 hours in, 22.9% (22/176) occurred in the second half-hour, and 15.6% (15/176) occurred in the first half-hour (Appendix Table 1).

### Injury Outcomes and Return to Play

Most injuries were nonoperative, as 97.6% (166/176) of injuries did not require surgery (Appendix Table 1). Although 83.1% (143/176) of injured athletes were able to resume activity, 14.0% (24/176) of athletes did not return to play. More than one fourth (28.5%, 49/176) of athletes returned within 1 to 2 days, 25.6% (44/176) returned after 3 to 6 days, whereas 4.1% (7/176) required more than 22 days to return (Table 4).

**Table 4:** Return to Play

	Male (N = 68)	Female (N = 108)	Total (N = 176)	*P* Value
Return to play				.323∗
N-Miss	1	3	4	
Returned in:				
<1 d	0 (0.0%)	2 (1.9%)	2 (1.2%)	
1-2 d	23 (34.3%)	26 (24.8%)	49 (28.5%)	
3-6 d	14 (20.9%)	30 (28.6%)	(44 (25.6%)	
7-9 d	10 (14.9%)	12 (11.4%)	22 (12.8%)	
10-21 d	9 (13.4%)	10 (9.5%)	19 (11.0%)	
>22 d	3 (4.5%)	4 (3.8%)	7 (4.1%)	
Did not return				
Medical disqualification	1 (1.5%)	1 (1.0%)	2 (1.2%)	
Athlete’s decision	1 (1.5%)	1 (1.0%)	2 (1.2%)	
Other	2 (3.0%)	3 (2.9%)	3 (2.9%)	

∗ Fisher exact test for count data.

## Discussion

The most important finding of this study is that the ankle, knee, and wrist are the most common locations for injuries seen in high school tennis players. The most common types of injuries were ligament sprains and muscle strains in the lower extremity. Injuries were also most attributed to overuse. Although few studies in the literature mention the context of injury, we note that most noncontact injuries occurred during general play or while chasing a ball. Fortunately, nearly one half of all athletes returned to play within a week of injury, and few required surgery.

In our study population consisting of American high school tennis players, we found the ankles and the knees to be the 2 most common body parts injured. This has been previously shown in other demographics, as well. For instance, one study involving 55 Swedish junior tennis players found lower-extremity injuries, representing 51% of all injuries, to be the most common location of tennis injuries.^10^ In contrast, other studies involving elite junior Australian tennis players and boys participating in the United States Tennis Association’s National Boys’ Tennis Championships both reported back injuries to be the most common.^9^^,^^11^ This difference in findings may be attributed to level of play. Because most participants in this study did not play tennis outside of school, they may have been less prone to overuse injuries involving the trunk compared to elite-level adolescent players, who may be playing at a greater intensity and/or frequency.

Our findings for injury type and injury mechanism were similar to those found in other studies of tennis athletes. For example, we found strains and sprains to be the most common injury types, with fractures and dislocations being rare. These results are similar to those of Dines et al.^9^ in youth and pediatric tennis players in the United States. In addition, we determined overuse to be the most common mechanism of injury. This result is echoed by findings from Johansson et al.^10^ of a significant increase in injury risk in relation to the volume of work and training their tennis athletes underwent. The relationship between overuse and injury has been further reiterated by Myers et al.,^19^ who found that athletes who underwent an acute increase in training load were at greater risk of getting injured.

Our study did not find any appreciable differences in injuries among male and female tennis players. The differences between male and female injuries may be attributed to the level of competition found in our population. We expect that the intensity of play and level of skill in school-sanctioned events and competitions may differ from those found in amateur and professional tennis. However, a review of the literature on sex-based differences in injuries among young tennis players have returned with disparate results. Several studies on elite, youth tennis players outside the United States found no significant injuries between male and female players.^11^^,^^20^ In contrast, a report on elite, junior players in Sweden found that male players suffered mainly from ankle injuries, whereas female players most commonly had knee injuries.^21^ Another study done in Spanish tennis academies also found that male players suffered more from lower-limb injuries, as compared to female players, who more commonly suffered trunk injuries.^22^

Injury outcomes are a critical topic in sports injury epidemiology studies, especially for adolescent athletes. Previous work on Australian junior tennis players reported greater injury severity for male players compared with female players, defined as a greater amount of time away from play.^11^ They reported 3.6 ± 0.6 days lost in male players compared with 1.1 ± 0.9 days lost in female players. Our study found that more than 50% of the injured tennis players in this study were able to return to play within 6 days from the injury, and only 2.2% of players required surgical treatment.

### Limitations

This study is not without limitations. It should be noted that population size was limited in our study because the HS RIO only contains data on tennis injuries reported from the 2014/2015-2017/2018 academic years at more than 100 participating high schools. Because of this limited time frame, the generalizability of our results may be affected. Owing to the limited sample size, limitations to the generalizability of this study are due to convenience sampling. Because of this, we were unable to calculate injury rates or ratios and make further inferences. In addition, since the HS RIO database relies on injury reporting from ATs at each high school across the nation, there may be reporting bias. For instance, under-reporting of injuries due to variability in judgment or record-keeping by each AT should be considered.

## Conclusions

We found no difference in injury patterns between male and female U.S. high school tennis athletes. The ankles, knees, and wrists were the most commonly injured areas in this population. The most common types of injuries were ligament sprains and muscle strains. Although many injuries were new, athletes rarely required surgery and returned to play. Finally, we found no difference in injury patterns between male and female high school tennis athletes.

## Figures and Tables

**Appendix Table 1:** Injury by Year, Level of Play, Injury by Time of Tennis Season, Injury Incidence, Injury Context, and Surgery Requirement

	Male (N = 68)	Female (N = 108)	Total (N = 176)	*P* Value
Year				.040∗
2014-2015	10 (14.7%)	25 (23.1%)	35 (19.9%)	
2015-2016	13 (19.1%)	34 (31.5%)	47 (26.7%)	
2016-2017	22 (32.4%)	19 (17.6%)	41 (23.3%)	
2017-2018	23 (33.8%)	30 (27.8%)	53 (30.1%)	
Level of play				.341†
Varsity	60 (88.2%)	85 (78.7%)	145 (82.4%)	
Junior varsity	7 (10.3%)	16 (14.8%)	23 (13.1%)	
Freshman	0 (0.0%)	1 (0.9%)	1 (0.6%)	
Combined (practice/competition)	1 (1.5%)	6 (5.6%)	7 (4.0%)	
Tennis season				.332†
N-Miss	1	0	1	
Preseason	11 (16.4%)	28 (25.9%)	39 (22.3%)	
Regular season	54 (80.6%)	78 (72.2%)	132 (75.4%)	
Postseason	2 (3.0%)	2 (1.9%)	3 (2.3%)	
New or recurrent injury				.733∗
N-Miss	0	2	2	
Recurrent injury	9 (13.2%)	16 (15.1%)	25 (14.4%)	
New injury	59 (86.8%)	90 (84.9%)	149 (5.6%)	
Athletic exposure				.310∗
Competition	33 (48.5%)	44 (40.7%)	77 (43.8%)	
Practice	35 (51.5%)	64 (59.3%)	99 (56.2%)	
Time of injury during practice				.133†
N-Miss	33	47	80	
First half hour	2 (5.7%)	13 (21.3%)	15 (15.6%)	
Second half hour	11 (31.4%)	11 (18.0%)	22 (22.9%)	
1-2 h into practice	14 (40.0%)	27 (44.3%)	41 (42.7%)	
>2 h into practice	2 (5.7%)	1 (1.6%)	3 (3.1%)	
Unknown	6 (17.1%)	9 (14.8%)	15 (15.6%)	
Time point in competition				.192†
N-Miss	39	68	107	
Warm-up	1 (3.4%)	0 (0.0%)	1 (1.4%)	
First set	4 (13.8%)	8 (20.0%)	12 (17.4%)	
Second set	12 (41.4%)	12 (30.0%)	24 (34.8%)	
Third set	2 (6.9%)	9 (22.5%)	11 (15.9%)	
Other	0 (0.0%)	2 (5.0%)	2 (2.9%)	
Unknown	10 (34.5%)	8 (22.5%)	19 (27.5%)	
General play	17 (27.4%)	35 (34.7%)	52 (31.9%)	
Other	2 (3.2%)	0 (0.0%)	2 (1.2%)	
Unknown	7 (11.3%)	9 (8.9%)	16 (9.8%)	
Chasing/running after/to hit ball	19 (30.6%)	28 (27.7%)	47 (28.8%)	
Forehand ground stroke/service return	2 (3.2%)	4 (4.0%)	6 (3.7%)	
One-handed backhand ground stroke/service return	3 (4.8%)	1 (1.0%)	4 (2.5%)	
Two-handed backhand ground stroke/service return	0 (0.0%)	2 (2.0%)	2 (1.2%)	
Forehand volley	0 (0.0%)	2 (2.0%)	2 (1.2%)	
Two-handed backhand volley	2 (3.2%)	0 (0.0%)	2 (1.2%)	
Serve	5 (8.1%)	8 (7.9%)	13 (8.0%)	
During what type of play/practice did injury occur				.086†
N-Miss	5	9	14	
Competition singles play	20 (31.7%)	29 (29.3%)	49 (30.2%)	
Competition doubles play	12 (19.0%)	13 (13.1%)	25 (15.4%)	
Singles play during practice	10 (15.9%)	19 (19.2%)	29 (17.9%)	
Doubles play during practice	3 (4.8%)	5 (5.1%)	8 (4.9%)	
Skills drills during practice	6 (9.5%)	24 (24.2%)	30 (18.5%)	
Other	1 (1.6%)	3 (3.0%)	4 (2.5%)	
Unknown	11 (17.5%)	6 (6.1%)	17 (10.5%)	
Site of competition/performance				.930†
N-Miss	36	64	100	
Home	18 (56.2%)	23 (52.3%)	41 (53.9%)	
Away	12 (37.5%)	19 (43.2%)	31 (40.8%)	
Neutral site	2 (6.2%)	2 (4.5%)	4 (5.3%)	
Did injury require surgery				.154†
N-Miss	1	5	6	
No	67 (100.0%)	99 (96.1%)	166 (97.6%)	
Yes	0 (0.0%)	4 (3.9%)	4 (2.4%)	

∗ Pearson χ^2^ test.

† Fisher’s exact test for count data.

## Supplementary Data

ICMJE author disclosure forms
